# Machine Learning for Cardiovascular Outcomes From Wearable Data: Systematic Review From a Technology Readiness Level Point of View

**DOI:** 10.2196/29434

**Published:** 2022-01-19

**Authors:** Arman Naseri Jahfari, David Tax, Marcel Reinders, Ivo van der Bilt

**Affiliations:** 1 Pattern Recognition and Bioinformatics Delft University of Technology Delft Netherlands; 2 Department of Cardiology Haga Teaching Hospital The Hague Netherlands

**Keywords:** mHealth, wearable, machine learning, cardiovascular disease, digital health, review, mobile phone

## Abstract

**Background:**

Wearable technology has the potential to improve cardiovascular health monitoring by using machine learning. Such technology enables remote health monitoring and allows for the diagnosis and prevention of cardiovascular diseases. In addition to the detection of cardiovascular disease, it can exclude this diagnosis in symptomatic patients, thereby preventing unnecessary hospital visits. In addition, early warning systems can aid cardiologists in timely treatment and prevention.

**Objective:**

This study aims to systematically assess the literature on detecting and predicting outcomes of patients with cardiovascular diseases by using machine learning with data obtained from wearables to gain insights into the current state, challenges, and limitations of this technology.

**Methods:**

We searched PubMed, Scopus, and IEEE Xplore on September 26, 2020, with no restrictions on the publication date and by using keywords such as “wearables,” “machine learning,” and “cardiovascular disease.” Methodologies were categorized and analyzed according to machine learning–based technology readiness levels (TRLs), which score studies on their potential to be deployed in an operational setting from 1 to 9 (most ready).

**Results:**

After the removal of duplicates, application of exclusion criteria, and full-text screening, 55 eligible studies were included in the analysis, covering a variety of cardiovascular diseases. We assessed the quality of the included studies and found that none of the studies were integrated into a health care system (TRL<6), prospective phase 2 and phase 3 trials were absent (TRL<7 and 8), and group cross-validation was rarely used. These issues limited these studies’ ability to demonstrate the effectiveness of their methodologies. Furthermore, there seemed to be no agreement on the sample size needed to train these studies’ models, the size of the observation window used to make predictions, how long participants should be observed, and the type of machine learning model that is suitable for predicting cardiovascular outcomes.

**Conclusions:**

Although current studies show the potential of wearables to monitor cardiovascular events, their deployment as a diagnostic or prognostic cardiovascular clinical tool is hampered by the lack of a realistic data set and proper systematic and prospective evaluation.

## Introduction

### Background

The use of diagnostic modalities in cardiovascular disease is often limited to hospital visits. As a result, the clinical value may be limited by the short observation period. This is especially problematic for cardiovascular problems that do not manifest constantly, such as paroxysmal arrhythmias, heart failure, or even chest discomfort that may not be present during the hospital visit. Advancements in eHealth, especially in wearable technology, such as electrocardiograms (ECGs) [1] and photoplethysmograms (PPGs) [2], and subsequent signal processing by machine learning have enabled new opportunities for remote monitoring in the outpatient setting.

Continuous monitoring over long periods has shown to be effective [3,4]. For example, remote monitoring of patients with cardiac diseases, using pacemakers or implantable cardioverter defibrillators and patients with heart failure have improved patient care [5]. However, current sensors used in health care, such as Holter devices, are limited to a maximum of 14 days (but typically endure 24 hours) of continuous monitoring, limiting the use of these devices. Overcoming this could enable early warning systems for acute events such as cardiac arrest and could capture subtle cardiovascular exacerbation or rehabilitation that manifests over a much longer time because of, for example, changes in lifestyle or intervention.

Although widely used, currently 24-hour ECG or blood pressure monitoring devices are cumbersome to wear and impose a burden on patients in a longitudinal setting. Rechargeable, easy-to-wear sensors, such as smartwatches, are becoming an interesting alternative as they contain sensors with a potentially unlimited observation period with minimal burden to the patient for a fraction of the costs. However, the signals that these wearables measure, such as the PPG-derived heart rate, activity, and skin temperature, are not clinically informative enough for clinical decision-making by a cardiologist. With current developments in artificial intelligence (AI), a powerful solution is expected from machine learning algorithms that can learn the relationship between the wearable sensor signals and a cardiovascular outcome in a (fully) data-driven manner.

Another great benefit of automatic cardiovascular diagnostics and prognostics by machine learning is minimizing inter- and intraobserver variability, which is a major problem in the subjective interpretation of clinical and diagnostic information by human cardiologists. Interobserver disagreement [6,7] because of, for example, differences in experience or specialization and intraobserver disagreement because of stress or fatigue [8], can be minimized. Variations in clinical practice may lead to medical errors, whereas automatic systems are not (or less) susceptible to such factors. Another possibility is to exclude patients who experience symptoms such as chest pain, which are not caused by cardiovascular disease. Automatic exclusion of these patients can reduce unnecessary visits to a cardiologist; relieving the cardiologist, thereby increasing the capacity of cardiovascular care; and directing patients to the proper specialist quicker.

Because of these promises, the field of research on diagnosing cardiovascular events from wearable data is very active and many machine learning solutions are being presented to automatically detect cardiovascular events. Various reviews have been presented to categorize the developed machine learning tools. A study by Krittanawong et al [9] shows that a plethora of wearable devices are researched for a variety of cardiovascular outcomes and discusses a paradigm for remote cardiovascular monitoring consisting of sensors, machine learning diagnosis, data infrastructure, and ethics. They conclude that especially the latter two aspects have several unaddressed challenges. An overview of wearable devices on the market is provided by Bayoumy et al [10]. The study reports their frequency of use in (cardiovascular) trials and Food and Drug Administration status. As reported by Giebel and Gissel [11], most mobile health devices for atrial fibrillation detection are not Food and Drug Administration approved and therefore cannot be used in cardiovascular monitoring systems.

### Objectives

Although many machine learning tools have been proposed and studies have shown good performance, they do not seem to have been implemented in operational and functional health care systems. Therefore, we decided to systematically review the machine learning tools to detect cardiovascular events from wearable data from the perspective of their technology readiness level (TRL), that is, how far these proposed tools are in realizing an operational system and what factor is impeding them to get there. The TRL paradigm originates from the National Aeronautics and Space Administration and is a way to assess the maturity level of a particular technology used in space travel by giving solutions a score from 1 to 9 in increasing order of readiness, from basic technology research (score 1) to launch operations (score 9) [12].

**Figure 1:**
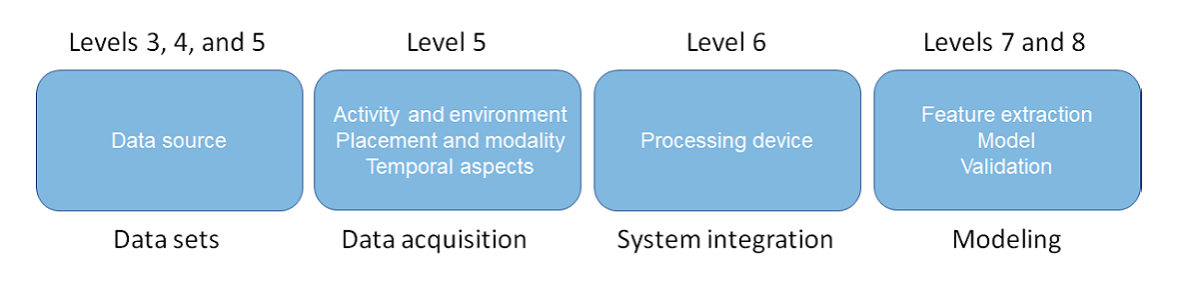
Taxonomy of the eligible studies. TRLs are based on the proposed descriptions for machine learning for medical devices proposed by Fleuren et al [14]. The studies were categorized according to the relevance of their content to these descriptions (aspects within boxes) and were grouped and assigned to the different TRLs (below and above boxes). TRL: technology readiness level.

Interestingly, 2 studies tailor the TRL framework for medical machine learning. A study by Komorowski [13] proposes a TRL for supervised, unsupervised, and reinforcement learning problems and describes criteria to reach TRLs 3, 4, 6, and 7. A description of the 9 TRLs for medical machine learning in intensive care medicine, including examples, is proposed by Fleuren et al [14]. We review the wearable-based cardiovascular machine learning solutions following the framework by Fleuren et al [14] adjusted for remote medicine. We identify aspects in the studies and systematically assign these to TRLs and group some of the TRLs together in a taxonomy to help interpret their relevance (Figure 1). We address the overuse of benchmark data sets, considerations on data acquisition related to the environment and type of sensor, integration in a health care system, construction of the machine learning model, and subsequent model validations.

By assessing current methods by their technological readiness, we show that the current methodologies are promising but that deployment is severely hampered by the lack of realistic data sets and proper systematic and prospective evaluation. To arrive at a readiness that is operational at the health care system level, these bottlenecks need to be resolved.

## Methods

### Screening

**Figure 2:**
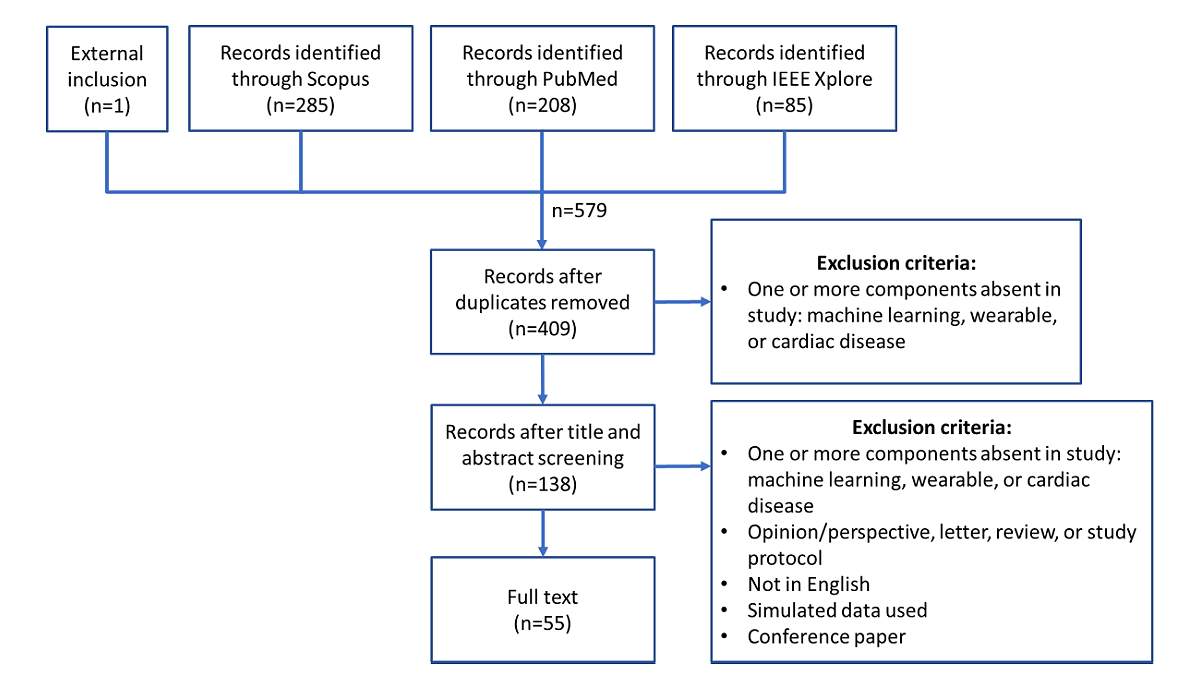
PRISMA (Preferred Reporting Items for Systematic Reviews and Meta-Analyses) flow diagram for the systematic review.

The systematic review was performed by following the PRISMA (Preferred Reporting Items for Systematic Reviews and Meta-Analyses) guidelines [15], as shown in Figure 2. We followed the patient or population, intervention, comparison, and outcomes framework for our research question, which was as follows: “In patients with cardiovascular disease, using machine learning with data from wearables, what methods and accompanying limitations are used, to deploy this technology to detect and predict cardiovascular disease in standard healthcare?”

### Study Inclusion

Search queries were performed on September 26, 2020, in the electronic databases Scopus, PubMed, and IEEE Xplore. Only peer-reviewed journals were considered. Studies were eligible for inclusion if data were acquired from wearables, a machine learning method was used, and had the goal to detect or predict cardiovascular disease (see Multimedia Appendix 1 for used queries). The following exclusion criteria were used: opinion or perspective, letter, review, study protocol, or conference paper; studies not in English; and studies in which only simulated data were used. The eligibility assessment was performed by the first author, ANJ. First, the title and abstract of each study were assessed for relevance based on the inclusion and exclusion criteria. The full texts of the remaining studies were then read and again subjected to the selection criteria. The second author, DT, verified this by reading a subsample of the selection.

### TRL and Taxonomy

From the eligible studies through discussions with all authors, the first author, ANJ, identified some general overarching evaluation aspects that the studies had in common and assigned these studies to a taxonomy (Multimedia Appendix 2 [16-70]). These aspects were related to one or more TRLs, as defined by Fleuren et al [14]. Accordingly, the eligible studies were assigned to the taxonomy and different TRLs (Figure 1). The TRL framework states that studies that use only a benchmark data set as a data source do not progress further than level 3. Furthermore, the framework originally grouped levels 3 and 4 together. We split these levels and assigned studies using their own acquired data without an external validation set from a different study level 4. Next, we assigned studies that use an external validation set from a different study to level 5; although, according to Fleuren et al [14], level 5 further requires that the acquired data set is realistic. However, we interpreted the independently acquired data representative of data recorded during the deployment of the machine learning system as realistic. Therefore, we differentiated levels 3, 4, and 5 mostly on the data sets being used for model deployment and related these levels to the data sets taxonomy. As level 5 mainly focuses on realistic data sets we also assigned practical aspects of the wearables to this TRL. Here, we differentiated the following three aspects: (1) which modality is being measured by the wearable and where on the body it is placed; (2) under which conditions data are measured, such as in the wild or in controlled environments; and (3) for how long data are recorded, that is, the temporal aspect of the acquired data. Level 6 required integrating the machine learning model into a health care system. Therefore, the device in which the model was integrated into was assigned to this level. Finally, levels 7 and 8 required demonstrating the model as a cardiovascular tool. Therefore, the model effectiveness and validation aspects were assigned to these levels. Levels 1, 2, and 9 were disregarded here because none of the papers fit into these categories.

## Results

### Article Identification

A total of 578 records were retrieved from electronic databases. After the removal of duplicates, 70.8% (409/578) of records remained. One was externally included as it fulfilled the inclusion criteria but was missed by the search query because it did not explicitly mention the term machine learning. As shown in Figure 1, these were further narrowed down during title or abstract screening, resulting in 23.9% (138/578) of records. Finally, after full-text reading, 9.5% (55/578) of records remained to be covered in this study.

We related each of the studies to different TRLs for machine learning methods (*Methods*) according to an identified taxonomy of different evaluation criteria that relate to these TRLs (Figure 1; *Methods*). The TRL framework states that studies that use only a benchmark data set do not progress further than level 3.

### Study Characteristics

The key characteristics of the eligible studies are summarized in Multimedia Appendix 2. Notably, of the 55 studies, 27 (49%) exclusively used benchmark data sets, which were all defined as benchmark studies. Furthermore, of the 55 included studies, 6 (11%) were published before 2018 and the remaining 49 (89%) were published thereafter. In the following sections, the study characteristics are described more closely based on the taxonomy.

### Activity and Environment (Level 5)

**Figure 3:**
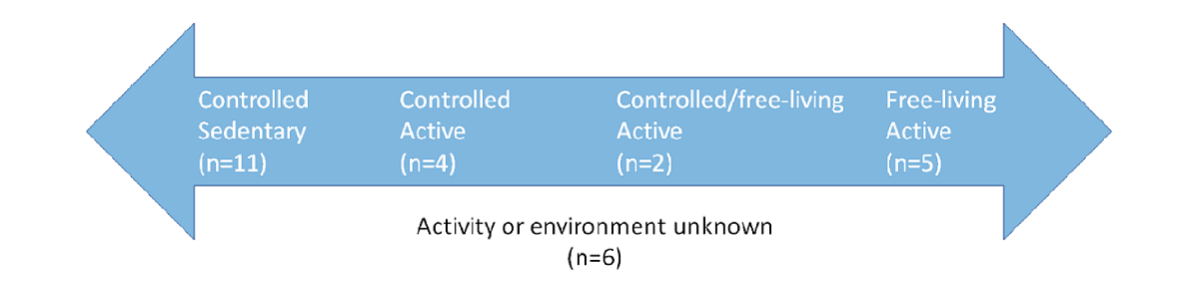
Studies ordered based on participant activity and acquisition environment. The leftmost scenario indicates highly controlled acquisition with sedentary participants. The opposite is described by the rightmost scenario where participants are monitored in an active, free-living situation. Controlled environment includes hospitals or laboratories. Free-living participants are monitored during their daily routines.

For studies that did not use benchmark data sets, they reported the data acquired either in a controlled environment (hospital or research laboratory) or in a free-living environment, where participants were remotely observed performing their natural daily routines. The latter is also known as *in-the-wild*. Furthermore, the activities of the participants can be divided into sedentary or active during data acquisition. To capture these two related aspects, we assigned studies on an axis representing a controlled environment and sedentary activity on one side and in-the-wild measurement of active participants on the other side of the axis (Figure 3). Interestingly, only 5 [16-20] studies mapped to the active, free-living situation that complied with the requirement of realistic data acquisition for these aspects that map to TRL5. Thus, only one-tenth of the studies used the potential of wearables to be used for remote, longitudinal monitoring.

### Placement and Modality (Level 5)

**Figure 4:**
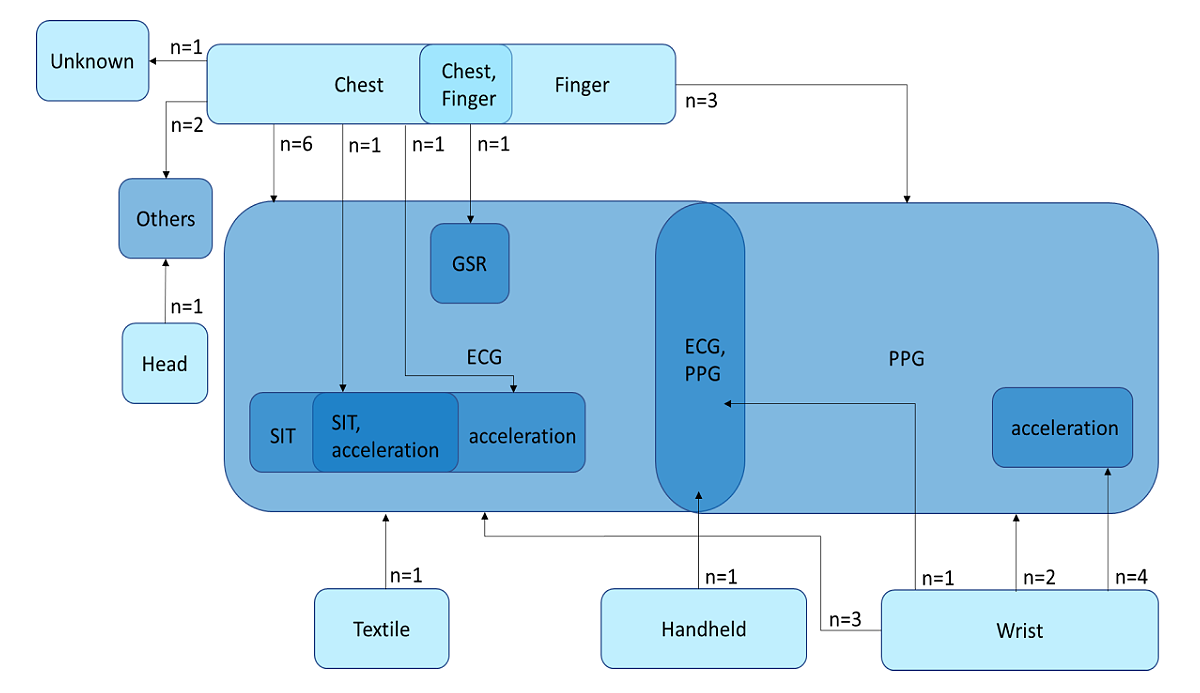
Placement and modalities of wearable sensors: light blue, placement of sensors; blue, modalities used. Others: head, near-infrared spectroscopy; chest, seismocardiography or gyrocardiography. Overlapping blocks represent multiple placements or modalities used. ECG: electrocardiogram; GSR: galvanic skin response; PPG: photoplethysmogram; SIT: skin impedance and temperature.

Realistic data acquisition requires continuous monitoring. Practically, the wearable should therefore not burden the participant when wearing. This burden depended mostly on the placement of the sensor on the body. In addition, the placement also restricted the type of biometric signals that could be measured, which was referred to as the modality. We categorized studies based on the placement and modality for the nonbenchmark studies jointly (Figure 4). The sensor placements for cardiovascular monitoring that results in the least burden for the patient, and thus would be the best candidates to acquire a realistic data set, were the wrist and finger. Less than half (N=13) of the studies were reported with such placements, of which 8 (62%) studies acquired one modality: 3 (23%) studies acquired wrist-based ECGs [18,21,22], 2 (15%) studies acquired wrist-based PPGs [17,23], and 3 (23%) studies acquired finger-based PPGs [24,30,37]. Of the 13 studies, the remaining 5 (39%) studies acquired wrist-based multimodal data: 4 (31%) studies acquired PPGs and accelerometer data [19,20,29,47] and 1 (8%) study acquired both ECGs and PPGs [25]. Thus, the wrist and finger severely limited the additional modalities that were measured (usually only acceleration), although wearables were shown to be able to measure increasing number of modalities [10].

### Temporal Aspects (Levels 5, 7, and 8)

**Figure 5:**
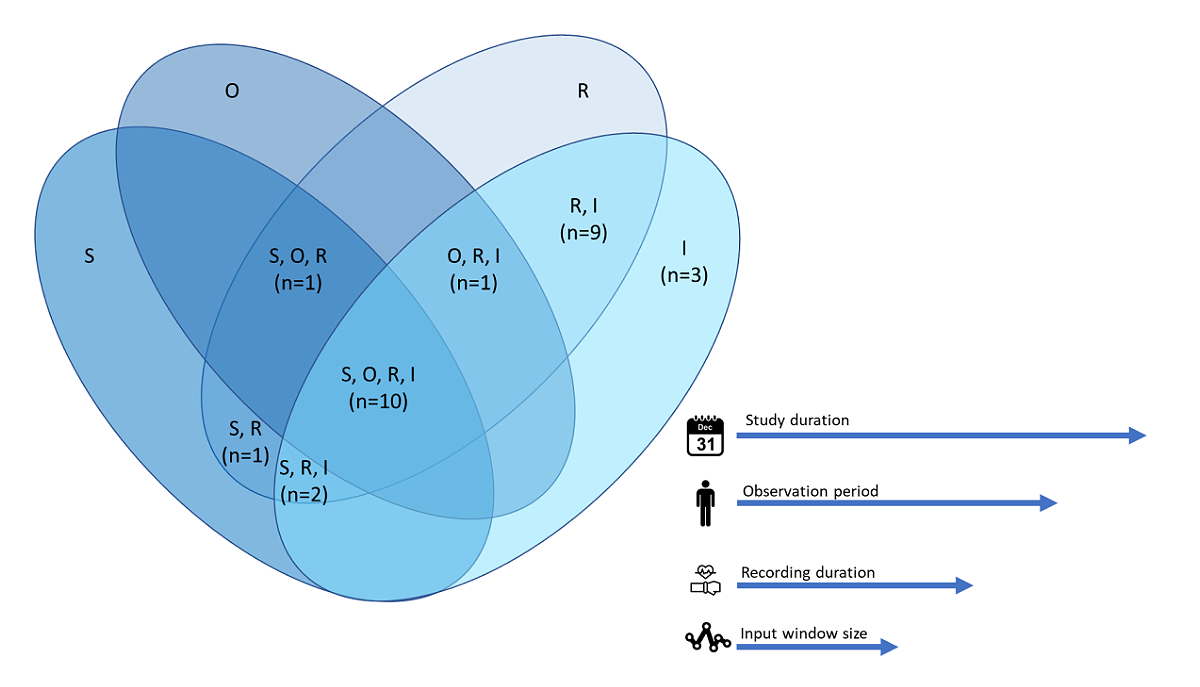
Venn diagram of reported temporal aspects described in the studies. The S, O, R, and I are represented in the legend. I: input window size; O: observation period; R: recording duration; S: study duration.

Besides the requirement of a realistic data set in level 5, levels 7 and 8 required phase 2 and phase 3 studies, respectively. In the context of drug testing, this requires an investigation of the effective, but safe, drug dosage. Analogously, for wearable machine learning, this translated to the time a participant must be exposed to a machine learning model before a cardiovascular outcome could be accurately detected or predicted. Therefore, a realistic deployment setting is dependent on the length of time participants are observed. As it is further essential to characterize the data for reproducibility and the description under which circumstances a model is valid, we decided to outline the temporal aspect of the acquired wearable data in more detail. We recognized the following four levels of time aspects: (1) study duration, (2) observation period, (3) recording duration, and (4) input window size (Figure 5). Within the study duration, patients were included and observed for a certain period—the observation period. The lengths of these periods had an impact on the realistic deployment of a system. For example, Quer et al [71] used wrist-worn Fitbit devices to show that resting heart rate within individuals had a significant seasonal trend in longitudinal data. Therefore, a model constructed using data from a certain period might not be valid for another period. It was therefore important to consider how long the participants were observed to ensure this seasonal effect was incorporated in the model. Within the observation period, the wearable recorded a time series. Theoretically, this could be as long as the observation period itself. However, patients could interrupt the measurements for several reasons (eg, to charge the device and low compliance rate). We denoted the duration of a continuously measured part of the time series as the recording duration. Finally, the records were further segmented into windows, from which features were generated or which were used as raw inputs to a machine learning model. We referred to the duration of these windows as the input window size (I).

We assessed the temporal aspects of all the nonbenchmark studies (Figure 5). One study did not report any aspects [26] and was omitted from the Figure 5. Another study used multiple fixed input window sizes to incorporate different timescales of the data [19]. Overall, most studies did not report all the aspects and were thus not comprehensive about their data characteristics. In almost all studies, the recording rate and input window size were reported, whereas the study and observation periods were mentioned in about half of the studies. For a realistic data set, required for level 5 and progression to level 7 or 8, the observation period and recording duration were specifically important, as we found in 12 studies. Three studies used an observation period of 24 hours [23,32,64]; one for a week [17], one for 2 weeks [27], and one for 90 days [16]. Overall, 2 studies implied an observation period of months but did not explicitly report it [19,20]. One considered recordings of at least eight hours [19] and one reported an average recording duration of 11.3 hours [20]. Finally, only one [27] fully used the potential of wearables and reported a (near-) continuous recording duration.

### Cardiovascular Outcomes (All Levels)

**Figure 6:**
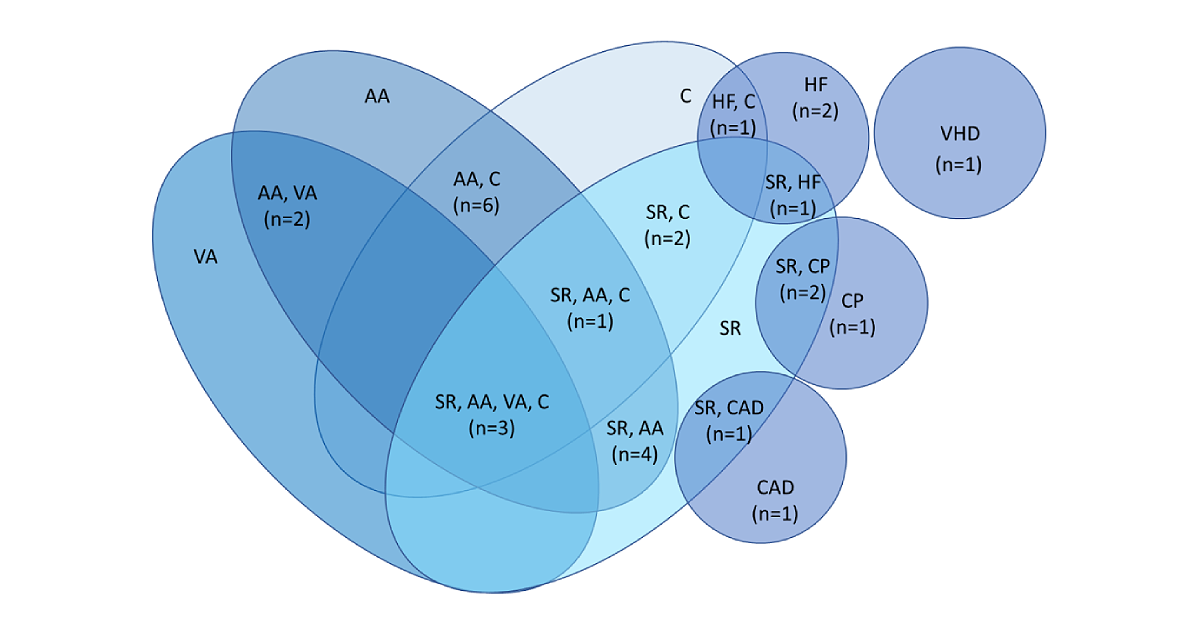
Studies categorized according to the type of cardiovascular outcomes predicted by the models. AA: atrial arrhythmia; C: control; CAD: coronary artery disease; CP: cardiovascular prevention; HF: heart failure; SR: sinus rhythm; VA: ventricular arrhythmia; VHD: valvular heart disease.

Although the required observation period and recording duration to detect or predict a cardiovascular outcome is still an open and active research topic, these periods will be different for different outcomes. Therefore, we inventoried which (combinations of) cardiovascular outcomes were considered in which studies (Figure 6). Interestingly, the control group was defined differently in each study. Only half of the nonbenchmark studies included a (normal) sinus rhythm class as control and could therefore exclude the presence of cardiovascular disease in participants. From these, 8 studies [17,21-23,28-31] used data from healthy individuals to represent normal sinus rhythm. The remaining 6 studies [32-37] derived normal sinus rhythm data from patients with arrhythmia (such as paroxysmal atrial fibrillation) or were unclear about the control group. Three studies had cardiovascular (disease) prevention as the target. One of these described this as a cardiovascular risk assessment where the predicted classes were healthy, precaution, and critical status [28]. Another study predicted vascular age and 10-year cardiovascular disease risk [34]. The third assigned a cardiorespiratory fitness score [27]. Notably, only the first 2 studies constructed a prognostic model. Two other prognostic models forecast cardiac arrest and heart failure exacerbation by forecasting rehospitalization after heart failure admission [16,21].

### Bottleneck TRL5

Although many cardiovascular outcomes were investigated with wearables, the promising studies that have reached level 5 were all focused on atrial arrhythmia using wrist-based PPGs. However, their temporal properties were often inconclusive, as they were not reported. Moreover, to progress to level 6, a model should be functional within a health care system (even if it was merely used observationally). None of the studies progressed to this level. An overview of the level 5 models, including the modalities that they are based on, is given in Table 1. Although none of the methodologies progressed to level 6, we decided to prospectively evaluate the studies to investigate the progression of the current state.

**Table 1:** Studies fulfilling requirements for technology readiness level 5.

Study	Outcomes	Modality	O^a^	R^b^	I^c^
Torres-Soto and Ashley [17]	Sinus rhythm, atrial arrhythmia	PPG^d^	1 week	NR^e^	25 seconds
Bashar et al [18]	Atrial arrhythmia, ventricular arrhythmia	ECG^f^	NR	NR	2 minutes
Tison et al [19]	Atrial arrhythmia, control	PPG, accelerometer^g^	NR	>8 hours a day	5 seconds, 30 seconds, 5 minutes, and 30 minutes
Wasserlauf et al [20]	Atrial arrhythmia, control	PPG, accelerometer	NR	11.3 hours a day	1 hour

^a^O: observation period.

^b^R: recording duration.

^c^I: input window size.

^d^PPG: photoplethysmogram.

^e^NR: not reported.

^f^ECG: electrocardiogram.

^g^Sensor-provided heart rate and step counter data.

### Processing Device (Level 6)

Integration in a health care system could be carried out on different devices. These studies demonstrated their models on either a computer (eg, a server), smartphone, or embedded device (Table 2). Only the latter two enabled real-time cardiovascular monitoring locally on the patient side, required for real-time detection and prevention of acute cardiovascular disease, as real-time information exchange to an external system would require high battery consumption and was therefore not feasible. Smartphones were used in both benchmark [38-40] and nonbenchmark [21,30,31,35] studies. Embedded devices, however, had only been demonstrated in benchmark studies [41-44].

**Table 2:** Processing device of trained models used in studies.

Processing device	Benchmarks included, n	Benchmarks excluded, n
Computer	44	24
Smartphone	7	4
Embedded device	4	0

### Feature Extraction Methods (Levels 7 and 8)

**Figure 7:**
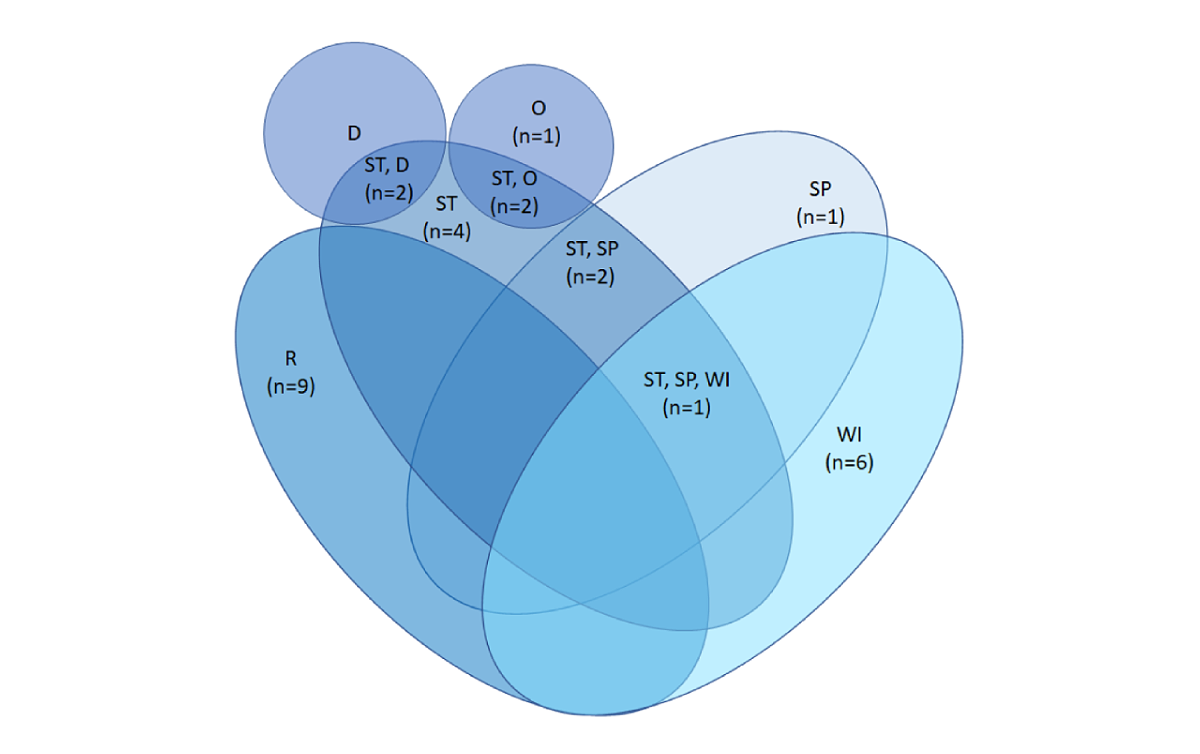
Features used in the studies. D: demographic; O: others; R: raw; SP: spectral; ST: statistical; WI: waveform information.

Levels 7 and 8 of the TRL assessed the model effectiveness through phases 2 and 3 clinical trials. We translated that to what features from the observed modalities were being used to construct the models. A significant number of studies used ECG as a modality and used different information from fiducial points [72] to extract features (Figure 7). In many studies, samples were selected before and after the R-peak. For example, the RR interval is the time interval between 2 adjacent R-peaks. Some studies also used techniques to locate other fiducial points and used the time interval between them as features [45]. Together, we denoted these types of features as waveform information features. Next to the specific ECG features, more general features could be derived, such as statistical features (eg, heart rate [variability] derived from 10 RR intervals) or spectral features obtained through techniques such as the Fourier transform. Raw data could also be used as features upon which a neural network can be used to automatically learn informative features [46]. Next to the features based on the sensed signal, demographic information could be used to provide more context [28,47]. Benchmark studies mostly use raw features (using the same data set) and were, therefore, excluded from this study. However, it is noteworthy that 2 of these used more advanced methods, namely, compressed learning [48] combined with dynamic time warping [49].

The most commonly used features were raw features (studies: 9/28, 32.1%). This was followed by waveform information and statistical features. In all, 2 studies also included demographic metadata from participants [28,47]. One study used hemoglobin parameters [26], which we represented in the *others* group in Figure 7. Interestingly, 1 study included timestamps [19]. From the 11 studies that used multimodal data (Figure 4), 6 (55%) studies extract features for each modality were separately extracted. Of the 11 studies, the remaining 5 (46%) studies exploited the covariance among the modalities in feature extraction, although 1 (9%) study did not elaborate on the exact method [16]. For example, of the 15 studies, 1 (9%) study computed the time between an R-peak in the ECG and the closest following peak in the PPG [34]. Of the 5 studies, 2 (40%) studies concatenated windows of the different modalities and then extracted the features [20,50] and 1 (20%) study concatenated windows whereafter a convolutional layer in a neural network is used to automatically extract features from the concatenated data [19].

### Model Construction Methods (Levels 7 and 8)

Another aspect that defines the model effectiveness relates to the type of models being constructed, which we categorized across both the benchmark and nonbenchmark studies (Table 3). Most of the studies used a neural network, and most of them were nonsequential (eg, convolutional and multilayer perceptron). A noteworthy type is the spiking neural network [51,52], which is designed to be energy efficient and suitable for real-time cardiovascular monitoring in an embedded device. Although sequential models were specifically designed for sequence or time series, these types of models were used much less. Some studies had combined sequential and nonsequential neural network architectures [17,19,32,42,46,53]. After the neural networks, most of the models were classical machine learning methods, including linear models: support vector machines; decision trees; and similarity-based models, such as k-nearest neighbor classifiers. Furthermore, ensemble methods had been used that combined multiple simpler models to construct a more complex model [22,28,44,50,54-56]. Finally, 2 studies used models that explicitly exploit the hierarchical structure of medical time series data: a hierarchical Bayesian model [27] and a Multiple-Instance Learning via Embedded instance Selection model [23].

**Table 3:** Types of machine learning models used in the studies.

Model type	Number of times used
Nonsequential	30
Classical	20
Ensemble	9
Sequential neural network	6
Nonsequential + sequential neural network	5
Hierarchical	2

### Validation (Levels 7 and 8)

The effectiveness of a model was heavily influenced by the number of samples with which the model had been trained. In phase 2 and phase 3 studies, a priori power analyses were performed to estimate the required sample size per group or class to observe an effect. It was empirically shown by Quintana [73] that for heart rate variability studies, an effect size of 0.25, 0.5, and 0.9 corresponded to a low, medium, and high effect, respectively. The corresponding sample sizes were 233, 61, and 21 for 80% statistical power and 312, 82, and 28 for a 90% statistical power. We considered nonbenchmark studies with a sufficient sample size per group or class, from which 9 studies remained. From the remaining 9 studies, a power of 90% was reached with small [19,20,24] and large [16,30,37,47] effect sizes, and 2 studies [29,32] achieved 80% power with a large effect size.

This showed that studies generally choose a train sample size (per group or class) that is too small to find a significant effect based on a priori power analysis.

**Figure 8:**
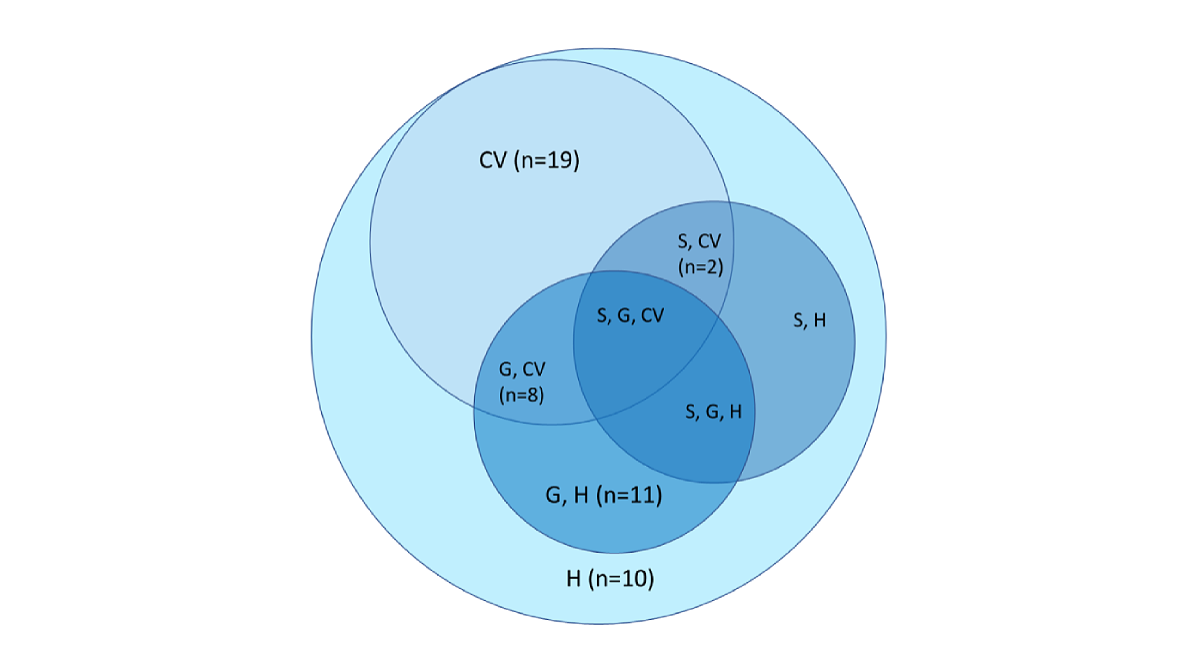
Venn diagram of validation methods used in the studies. CV: cross-validation; G: grouping; H: holdout; S: stratification.

In contrast to a priori power analysis, the purpose of model validation is to retrospectively analyze the performance of the model on data it has not seen before, that is, to assess the generalization error of the model. The included studies chose from 2 validation schemes: cross-validation and holdout [74] (Figure 8), although 5 studies [16,20,28,64,65] did not report the validation method. When splitting data into training and testing, one needed to ensure nonoverlapping grouping and stratification of the data (Figure 8). With nonoverlapping grouping [75], one ensured that the same group of data did not appear in both the training and test sets, for example, avoiding that data from the same participant was in both the training and test set, albeit the samples might be from different periods. With stratification, one ensured that both the training samples and the test samples exhibit a similar proportion of samples for an arbitrary variable. For example, it was important to keep the proportion of men and women consistent or to ensure that the proportion of sensor samples representing normal rhythm and arrhythmia is equal. For progressing to TRL 7, 4 studies used leave-one-subject-out group cross-validation [18,23,27,45] and 4 other types of group cross-validation [29,30,37,44]. Ideally, a stratified group cross-validation is used, but none of the studies used this. In addition to validation strategies, it is important to use replication data, that is, completely independently acquired data, which was only done in 11 [17,18,21,24,25,31,33,35,36,40,70] studies.

It is important to realize that data sets could suffer from highly imbalanced classes. An example is when there are proportionally more samples representing sinus rhythm than atrial fibrillation. In this case, the model may be biased to focus more on correctly classifying sinus rhythm, as this contributed more to higher overall classification performance. However, this led to poor characterization of cardiovascular disease, as the corresponding samples would be misclassified more often than sinus rhythm. In all, 6 studies [32,41,59-62] mitigated this by (randomly) up-sampling the minority class. A total of 4 studies [22,29,48,52] used the synthetic minority oversampling technique [76].

Finally, it is noteworthy that some studies [41-43,45,49,51,63] constructed a semi–patient-specific model. This could be beneficial, as there were large differences in heart rate data among individuals [71]. This was done by training only a small number of samples from the target patient together with data from other patients. The test set consisted of the remainder of the target patient’s samples, which caused overlapping grouping between the training and test sets.

## Discussion

### Principal Findings

We have shown that machine learning–based technologies that detect cardiovascular outcomes using wearables, bottleneck at TRL5, most dominantly on the requirement of proper realistic data acquisition. To progress to the next level of technology readiness, models need to become operational (either interventional or observational) in a health care system. A study by Komorowski [13] supports these observations and defines the lack of testing or deployment in clinical practice as an *information bottleneck*, which often occurs in medical machine learning. Moreover, half of the eligible studies used a benchmark data set (27/55, 49%), and the most common data set [77] was used 18 times. We argue that overusing a data set can introduce bias and overfitting, effectively making such a data set useless, thereby increasing the need for realistic data sets even more.

The usefulness of wearable cardiovascular diagnostics lies in free-living and active situations because the low burden for wearing them and the 24/7 monitoring abilities. Placement of the sensor on the wrist does fit these criteria best. Moreover, commercial-grade smartwatches can measure multimodal data with low battery consumption. This makes these types of sensors promising to use wearable technology for cardiovascular diagnostics. However, most studies do not fully demonstrate this potential. Moreover, very few prognostic models have been proposed so that cardiovascular disease prevention using wearable machine learning is, in fact, not (yet) well researched.

Although most studies include detailed baseline characteristics of the study population, it is worrisome that the data were not described with a similar level of consistency, structure, and detail. For example, some studies (explicitly or implicitly) have reported acquiring continuous wearable data, but participants do need to take off the device for charging or otherwise have a low compliance rate. These studies then fail to report these details; thus, it is unknown how *continuous* the data, that is, the length of the recording duration, actually is. We believe that, analogous to the baseline characteristics, data characteristics should be reported in detail to predict how a model will generalize when deployed in a particular setting and environment.

The segmentation of the time series data in the windows was performed with a fixed window size in all studies. None of the studies have considered a variable-length or adaptive window size. Furthermore, no previous physiological knowledge has been used to determine informative timescales. For example, the exercise-recovery curve (usually obtained from an exercise tolerance test) is often used to quantify cardiovascular characteristics during activity. This describes a participant’s ability to adaptively increase the heart rate during exercise and recover it back to a resting level after exercise. Studies that had access to accelerometer data did not look at similar timescale events. To this end, we believe that identifying informative timescales within the time series and incorporating this in the model can be valuable to detect cardiovascular diseases.

Remarkably, studies primarily prefer nonsequential neural networks over sequential ones, although the latter is designed for time series data. Similarly, the hierarchical structure of the data has rarely been exploited in the published models. We advocate that much more emphasis should be on the exploration of these models, although this also requires larger data sets as these methods are data hungry.

Although some studies make use of a healthy control group, most do not include a group with *no arrhythmia*, *sinus rhythm*, or a similar group, although diagnosing a participant having no arrhythmia at all is just as, or even more powerful, than detecting a specific heart problem. From a machine learning point of view, this can be seen as a one-class classification (outlier detection) problem: instead of predicting a diverse set of clinical outcomes, the focus of these models lies in modeling the *normal class* as good as possible and consider deviating data as abnormal. Thus, this would be an interesting avenue to explore. In general, it is important to have clearly defined data annotations. For example, some studies have annotated sinus rhythm events in patients with arrhythmia. One might question whether this is similar to annotated sinus rhythm events for nonarrhythmic individuals and whether a machine learning–based approach might fail by mixing these annotations.

We have shown that studies use a training sample size that is too small according to a priori power analysis. Sample size determination in machine learning [78] is focused on posthoc methods, such as learning curves [79]. Prehoc methods, such as power analysis, are difficult in machine learning, as there are many factors that influence the effect size of the model. Furthermore, we have discussed different validation schemes that can be used. An important observation is that a significant number of studies do not validate their model using a nonoverlapping grouping strategy. We believe that validation based on nonoverlapping grouping is crucial for cardiovascular machine learning and any medical machine learning validation in general. Without, experiments will simply suggest performances that are too optimistic.

We have shown that only a few papers used multimodal data and even less considered features across modalities. In our view, this is a missed opportunity; there is valuable information to be extracted when combining features from different modalities. An example is the correlation between heart rate and activity. When the heart rate changes abruptly without activity, this can indicate an interesting segment for a model to detect heart problems. As another example, 1 study used timestamps as features that can provide information about seasonality in longitudinal data. This could be used to inspect (change in) circadian rhythm as a biomarker for cardiovascular disease. Interestingly, ECG morphology is well researched and used as a feature. However, no analogous decomposition of PPG signals is used in the studies. Therefore, we advocate a similar exploration of the PPG signals.

Finally, we argue that in addition to the technical shortcomings discussed, societal factors (under the umbrella term ethical or socially responsible AI) must also be addressed [80]. From the patients’ point of view, there are concerns regarding reliability, privacy, and especially fairness and AI bias of the system [81]. Our findings of the lack of realistic data and the imbalance in data link to the latter, as it introduces sampling bias [82], for example. A study by Parikh et al [83] refers to this as a statistical bias and argues that, especially in the medical field, there can also be social biases that are caused by inequity of patients’ access to health care (technology) or a combination of both, for example, missing data in certain subgroups. Efforts should be made to remove bias in data (before exposing to an AI model) [80] and in the model itself. This referred to as *debiasing* [80,82,84].

From the physicians’ point of view, the performance of machine learning models is potentially reaching that of health care professionals’ point of view [85,86], which brings techno-dystopic fear of rivalry between AI and human experts. The study by Di Ieva [87] offers an alternative view by stating that this fear can be overcome by considering the success of multidisciplinary teams in modern medicine and that in line with that paradigm, AI is an assisting expert in that team, rather than a competitor.

As a final note, we would like to emphasize that we did not fully perform a quality assessment of the risk of bias in the clinical data acquisition of the studies. Instead, we used the TRL to capture these risks from a machine learning perspective and describe these limitations throughout. To this end, studies with low methodological quality did not achieve a higher TRL. In addition, we did not consider conference papers as journal papers are more comprehensive and elaborate in general. However, in the field of machine learning, conferences are used to publish completed research (not limited to an abstract as in other fields). Therefore, we might have missed new developments from conference papers that have been described in detail, yet not fully scrutinized as in journal papers.

### Conclusions

TRL has enabled us to perform a structured assessment of the (required) progression of machine learning–based wearable technology for deployment in an operational setting. We discussed that the promise is mainly achieved by acquiring longitudinal data from participants in a free-living environment, which is made possible because of low–energy-consuming sensors that are easy to wear. However, we have also observed that none of the studies detect or predict cardiovascular outcomes on realistic data, which limits TRL of this technology. In addition, we identified many other aspects that hamper deployment progression, which need to be addressed before the promise of using wearable technology for cardiovascular disease detection and prevention becomes reality. On the other hand, of the 55 included studies, 6 (11%) were published before 2018 and the remaining 49 (89%) after. Therefore, we expect a large increase in research popularity in the coming years.

## Appendix

Multimedia Appendix 1 Search queries performed in the three electronic databases.

Multimedia Appendix 2 Tables of study characteristics.

## Abbreviations

AI: artificial intelligence
ECG: electrocardiogram
PPG: photoplethysmogram
PRISMA: Preferred Reporting Items for Systematic Reviews and Meta-Analyses
TRL: technology readiness level
