# Supplementary material for: Machine Learning for Cardiovascular Outcomes From Wearable Data: Systematic Review From a Technology Readiness Level Point of View
Source: JMIR Med Inform. 2022 Jan 19;10(1):e29434. doi: 10.2196/29434 (PMC8811688; doi:10.2196/29434)
Supplement: Multimedia Appendix 1 [file medinform_v10i1e29434_app1.docx]

## Supplement 1

Table 1. Search queries performed in the three electronic databases.

| **Database** | **Search query** |
| --- | --- |
| Scopus | TITLE-ABS-KEY((”machine learning” OR ”deep learning”OR ”neural |
|  | networks” OR ”artificial intelligence”)AND (wearable OR |
|  | smartwatch OR ”fitness tracker”)AND (cardiovascular OR |
|  | cardiology OR cardiac OR heartOR ”atrial fibrillation” OR ”heart |
|  | failure” OR arrhythmia))AND (LIMIT-TO ( SRCTYPE,”j” ))AND |
|  | (LIMIT-TO(PUBSTAGE,”final”))AND |
|  | (LIMIT-TO(DOCTYPE,”ar”))AND (LIMIT-TO(LANGUAGE,”English”)) |
| PubMed & IEEE Xplore | ((”machine learning” OR ”deep learning” OR ”neural networks”OR |
|  | ”artificial intelligence”)AND (wearable OR smartwatch OR ”fitness |
|  | tracker”)AND (cardiovascular OR cardiology OR cardiac OR heartOR |
|  | ”atrial fibrillation” OR ”heart failure” OR arrhythmia) |
