# Supplementary material for: Machine Learning for Cardiovascular Outcomes From Wearable Data: Systematic Review From a Technology Readiness Level Point of View
Source: JMIR Med Inform. 2022 Jan 19;10(1):e29434. doi: 10.2196/29434 (PMC8811688; doi:10.2196/29434)
Supplement: Multimedia Appendix 2 [file medinform_v10i1e29434_app2.docx]

## Supplement 2

NR = Not Reported

Table 1. Benchmark, sample size, processing and TRL.

| **Study** | **Benchmark** | **Sample size** | **Processing** | **TRL** |
| --- | --- | --- | --- | --- |
| [1] |  | 50 | Computer | 4 |
| [2] | yes | 124 | Computer | 5 |
| [3] |  | 401 | Computer | 4 |
| [4] | Yes | 104 | Computer | 5 |
| [5] |  | 30 | Computer | 4 |
| [6] |  | 501 | Computer | 4 |
| [7] | Yes | 330 | Smartphone | 5 |
| [8] | Yes | 104 | Computer | 5 |
| [9] |  | 30 | Smartphone | 4 |
| [10] |  | 85 | Computer | 4 |
| [11] | Yes | NR | Computer | 5 |
| [12] |  | 46 | Computer | 4 |
| [13] |  | 40 | Computer | 4 |
| [14] |  | 100 | Computer | 4 |
| [15] |  | 3777 | Computer | 5 |
| [16] |  | 75 | Computer | 4 |
| [17] |  | 49 | Computer | 4 |
| [18] |  | 97 | Computer | 4 |
| [19] |  | 70 | Computer | 4 |
| [20] |  | 100 | Smartphone | 4 |
| [21] | Yes | 487 | Computer | 5 |
| [22] |  | 83 | Computer | 5 |
| [23] |  | 368 | Smartphone | 5 |
| [24] |  | 220 | Computer | 5 |
| [25] | Yes | 52 | Computer | 5 |
| [26] |  | 45 | Computer | 4 |
| [27] |  | 8299 | Computer | 5 |
| [28] |  | 7524 | Computer | 5 |
| [29] | Yes | 43 | Embedded | 3 |
| [30] | Yes | NR | Computer | 3 |
| [31] | Yes | 44 | Embedded | 3 |
| [32] | Yes | 47 | Computer | 3 |
| [33] | Yes | NR | Computer | 3 |
| [34] | Yes | 47 | Smartphone | 3 |
| [35] | Yes | NR | Smartphone | 3 |
| [36] | Yes | NR | Computer | 3 |
| [37] | Yes | 181 | Computer | 3 |
| [38] | Yes | NR | Computer | 3 |
| [39] | Yes | 23 | Computer | 3 |
| [40] | Yes | 47 | Computer | 3 |
| [41] | Yes | NR | Computer | 3 |
| [42] | Yes | 47 | Computer | 3 |
| [43] | Yes | 47 | Computer | 3 |
| [44] | Yes | 43 | Computer | 3 |
| [45] | Yes | 47 | Computer | 3 |
| [46] | Yes | 43 | Computer | 3 |
| [47] | Yes | 43 | Embedded | 3 |
| [48] | Yes | NR | Computer | 3 |
| [49] | Yes | NR | Computer | 3 |
| [50] | Yes | 290 | Computer | 3 |
| [51] | Yes | 44 | Computer | 3 |
| [52] | Yes | NR | Smartphone | 3 |
| [53] | Yes | 104 | Embedded | 3 |
| [54] | Yes | 44 | Computer | 3 |
| [55] | Yes | NR | Computer | 3 |

Table 2. Study duration, observation period and recording duration

| **Study** | **Study Duration** | **Observation Period** | **Recording Duration** |
| --- | --- | --- | --- |
| [1] | Mar 19 - Oct 2019 | 24H | 24H |
| [2] | Feb 17 - Apr 18 | 24H | 17H |
| [3] | May 19 - Jun 19 | NR | 3M |
| [4] | NR | NR | 30M |
| [5] | Jun 7, 17 | 22M | 22M |
| [6] | NR | NR | 1M |
| [7] | NR | NR | NR |
| [8] | NR | NR | 30M |
| [9] | NR | NR |  |
| [10] | NR | NR | 30S |
| [11] | NR | NR | 5M |
| [12] | NR | 14D | near continuous |
| [13] | NR | 5.7M | 5.7M |
| [14] | Aug 15 - Dec 16 | 90D | NR |
| [15] | train: Mar 13 - Jan 18 | NR | median 10.5H |
| [16] | Sep 17 - Apr 18 | 30m | 15M |
| [17] | NR | NR | NR |
| [18] | Oct 15 - Mar 16 | 5m | 5M |
| [19] | Mar 15 - Nov 15 | 10m | 10M |
| [20] | 18 - 19 | 15m | 15M |
| [21] | NR | NR | train: (1,2), test:60 R-R intervals |
| [22] | PIONEER-HCM: Aug 16 - Nov 17, MYK-491-001: Jan016 - Nov 18 | 1D | 5M |
| [23] | NR | NR | 16S |
| [24] | NR | train: 45min, test:1 week | NR |
| [25] | NR | NR | NR |
| [26] | NR | NR | 6M |
| [27] | Feb 16 - Mar 17 | NR | >8H per day |
| [28] | train: NR, test: May 17 - Sep 17 | NR | 11.3H |
| [29] | 1975 - 1979 | NR | 30M |
| [30] | NR | NR | 30M |
| [31] | 1975 - 1979 | NR | 30M |
| [32] | 1975 - 1979 | NR | 30M |
| [33] | NR | NR | 10H |
| [34] | 1975 - 1979 | NR | 30M |
| [35] | NR | NR | 3H |
| [36] | NR | NR | 30M |
| [37] | NR | NR | 1M |
| [38] | NR | NR | 1M |
| [39] | 1975 - 1979 | NR | 10H |
| [40] | 1975 - 1979 | NR | 30M |
| [41] | NR | NR | 1M |
| [42] | 1975 - 1979 | NR | 30M |
| [43] | 1975 - 1979 | NR | 30M |
| [44] | 1975 - 1979 | NR | 30M |
| [45] | 1975 - 1979 | NR | 30M |
| [46] | 1975 - 1979 | NR | 30M |
| [47] | 1975 - 1979 | NR | 30M |
| [48] | NR | NR | 2M |
| [49] | NR | NR | 1M |
| [50] | NR | NR | 2M |
| [51] | 1975 - 1979 | NR | 30M |
| [52] | NR | NR | NR |
| [53] | NR | NR | NR |
| [54] | 1975 - 1979 | NR | 30M |
| [55] | NR | NR | 3H |

Table. 3 Activity, setting, placement and modality. C = Controlled, F = Free-living,

A = Active, S = Sedentary.

| **Study** | **Environment** | **Setting** | **Placement** | **Modality** |
| --- | --- | --- | --- | --- |
| [1] | C | A | Chest | ECG |
| [2] | C | NR | Chest | ECG |
| [3] | C | S | wrist | PPG, ECG |
| [4] | NR | NR | Textile | ECG |
| [5] | C | A | Chest, finger | ECG, Galvanic Skin Response |
| [6] | C | S | Handheld | PPG, ECG |
| [7] | C | A | Chest | ECG |
| [8] | NR | NR | Chest | ECG |
| [9] | NR | NR | Wrist | ECG |
| [10] | C | S | Wrist | ECG |
| [11] | NR | NR | NR | ECG |
| [12] | CF | A | Chest | ECG, accelerometer, location |
| [13] | C | S | Chest | seismocardiogram, gyro-cardiogram |
| [14] | F | A | Chest | ECG, accelerometer, skin impedance & temperature |
| [15] | C | S | Finger | PPG |
| [16] | C | S | Finger | PPG |
| [17] | C | A | Head | near infrared spectroscopy |
| [18] | C | S | wrist | PPG, accelerometer |
| [19] | C | S | Wrist | PPG, accelerometer |
| [20] | C | S | Finger | PPG |
| [21] | C | S | Chest | ECG |
| [22] | C | S | Wrist | PPG |
| [23] | NR | NR | Chest | ECG |
| [24] | F | A | Wrist | PPG |
| [25] | F | A | Wrist | ECG |
| [26] | CF | A | chest | seismocardiogram |
| [27] | F | A | Wrist | PPG, accelerometer |
| [28] | F | A | Wrist | PPG, accelerometer |
| [29] | C | NR | Chest | ECG |
| [30] | C | NR | Chest | ECG |
| [31] | C | NR | Chest | ECG |
| [32] | C | NR | Chest | ECG |
| [33] | NR | NR | Chest , handheld | ECG |
| [34] | C | NR | Chest | ECG |
| [35] | NR | NR | Chest | ECG |
| [36] | NR | NR | Chest | ECG |
| [37] | NR | NR | Chest | ECG |
| [38] | NR | S | Handheld | ECG |
| [39] | C | NR | Chest | ECG |
| [40] | C | NR | Chest | ECG |
| [41] | NR | S | Handheld | ECG |
| [42] | C | NR | Chest | ECG |
| [43] | C | NR | Chest | ECG |
| [44] | C | NR | Chest | ECG |
| [45] | C | NR | Chest | ECG |
| [46] | C | NR | Chest | ECG |
| [47] | C | NR | Chest | ECG |
| [48] | NR | NR | Chest | ECG |
| [49] | NR | S | Handheld | ECG |
| [50] | C | NR | Chest | ECG |
| [51] | C | NR | Chest | ECG |
| [52] | NR | NR | Chest | ECG |
| [53] | NR | NR | NR | ECG |
| [54] | C | NR | Chest | ECG |
| [55] | NR | NR | Chest | ECG |

Table 4. Input window size, model, features and validation

| **Study** | **Input window size** | **Model** | **Features** | **Validation** |
| --- | --- | --- | --- | --- |
| [1] | 10S | CNN | WI | NR |
| [2] | variable, 3-beat units | CNN-RNN | R | GH |
| [3] | NR | Residual neural network | R | GH |
| [4] | 0.55S | CNN | R | GH |
| [5] | 10S | MLP, logistic regression, random forest | St, D | NR |
| [6] | variable, one beat | linear regression, decision tree, MLP | WI | GH |
| [7] | 30S | CNN | R | CV |
| [8] | 0.55S | Autoencoder | WI | CV |
| [9] | 5S | Decision tree | WI | GH |
| [10] | 30S | ensemble | St | CV |
| [11] | 30S | LSTM | WI | GH |
| [12] | 15S | Hierarchical Bayesian model | St, Sp | GCV |
| [13] | 10S | Decision tree, random forest, MLP | St, Sp | CV |
| [14] | 1M | Graph similarity | St, O | NR |
| [15] | 30S | SVM, CNN | R | CV |
| [16] | 30S | CNN, RNN, SVM | R | GCV |
| [17] | NR | MLP | O | GH |
| [18] | 5m | SVM | St, D | CV |
| [19] | 2m | kNN | St | GCV |
| [20] | 30S | CNN | R | GCV |
| [21] | train: (1,2) test:60 RR intervals | SVM | St | CV |
| [22] | 1 beat | MILES | WI | GCV |
| [23] | NR | MLP | R | GH |
| [24] | 25S | CNN-autoencoder | R | H |
| [25] | 2M | SVM, random forest | WI, St, Sp | GCV |
| [26] | 1s | Graph similarity | Sp | NR |
| [27] | 5s, 30s, 5m, 30m | CNN-LSTM | St, O | GH |
| [28] | 1H | CNN | St | NR |
| [29] | 2 RR intervals | MLP | WI | CV |
| [30] | 0.24S | Echo state network | WI | CV |
| [31] | 1 RR interval | CNN-LSTM | R | H |
| [32] | 0.22S | SVM | WI | GCV |
| [33] | 30S | Gradient boosted decision tree | WI, St | CV |
| [34] | 1 beat | MLP | WI | CV |
| [35] | 2S | MLP | WI, St | H |
| [36] | 25 RR intervals | MLP | WI | H |
| [37] | 1M | Decision Table, MLP, random forest kNN, logistic regression, SVM, ensemble | WI | CV |
| [38] | 1M | SVM, bagging trees | WI, Sp | CV |
| [39] | 1S | CNN | R | H |
| [40] | 1 P-QRS-T complex | SVM | R | CV |
| [41] | 9S, 15S | CNN | Sp | SCV |
| [42] | 0.7S around R-peak (0.25 left, 0.45 right) | spiking neural network | R | H |
| [43] | 0.8S around R-peak (0.4 left, 0.4 right) | CNN | R | SCV |
| [44] | 0.5S around R-peak(0.25 left, 0.25 right) | spiking neural network | R | GH |
| [45] | 1.1S around R-peak (0.37 left, 0.74 right) | MLP | R | H |
| [46] | 0.56S | LSTM | R | H |
| [47] | 0.7S around R-peak (0.25 left, 0.45 right) | LSTM | R | H |
| [48] | 1707ms | CNN-LSTM | R | CV |
| [49] | 5S | CNN | R | CV |
| [50] | 0.6S around R-peak (0.2 left, 0.4 right) | CNN-LSTM | R | CV |
| [51] | 0.5S around R-peak | CNN | R | GH |
| [52] | 20S | MLP | WI, St | CV |
| [53] | 0.65S around R-peak (0.25 left, 0.4 right) | Random forest | WI, Sp | GCV |
| [54] | 0.36S around R-peak | CNN | R | H |
| [55] | 0.2S around R-peak (0.75 left, 0.125 right) | Echo state network | WI | CV |

Table 5. Type of cardiovascular disease used as target condition. AA=Atrial Arrhythmia, VA=Ventricular Arrhythmia, C=Control, SR=Sinus Rhythm, HF=Heart Failure, CP=Cardiovascular Prevention, CAD=Coronary Artery Disease, VHD=Valvular Heart Disease.

| **Study** | **Clinical outcome** |
| --- | --- |
| [1] | AA, C |
| [2] | SR, AA, VA, C |
| [3] | AA, C |
| [4] | SR, AA, VA, C |
| [5] | SR, CP |
| [6] | SR, CP |
| [7] | SR, AA, VA, C |
| [8] | AA, VA |
| [9] | SR, CAD |
| [10] | SR, AA |
| [11] | SR, C |
| [12] | CP |
| [13] | VHD |
| [14] | HF |
| [15] | AA, C |
| [16] | SR, AA |
| [17] | CAD |
| [18] | HF, C |
| [19] | SR, AA, C |
| [20] | SR, AA |
| [21] | AA, C |
| [22] | SR, HF |
| [23] | SR, C |
| [24] | SR, AA |
| [25] | AA, VA |
| [26] | HF |
| [27] | AA, C |
| [28] | AA, C |
| [29] | SR, AA, VA, C |
| [30] | SR, AA, VA, C |
| [31] | SR, AA, VA, C |
| [32] | AA, VA |
| [33] | SR, AA, C |
| [34] | SR, VA, C |
| [35] | SR, AA, VA |
| [36] | SR, AF |
| [37] | SR, AA |
| [38] | SR, AA, C |
| [39] | SR, AA, C |
| [40] | SR, AA, VA, C |
| [41] | SR, AA, C |
| [42] | SR, VA, C |
| [43] | SR, VA, C |
| [44] | SR, AA, VA, C |
| [45] | SR, AA, VA, C |
| [46] | SR, AA, VA |
| [47] | SR, AA, VA, C |
| [48] | SR, CAD, C |
| [49] | SR, AA, C |
| [50] | SR, CAD |
| [51] | SR, AA, VA, C |
| [52] | VA, C |
| [53] | SR, CAD |
| [54] | AA, VA |
| [55] | VA, C |

1. Lai D, Bu Y, Su Y, Zhang X, Ma CS. A Flexible Multilayered Dry Electrode and Assembly to Single-Lead ECG Patch to Monitor Atrial Fibrillation in a Real-Life Scenario. IEEE Sens J 2020; [doi: 10.1109/JSEN.2020.2999101]

2. Jeon E, Oh K, Kwon S, Son HG, Yun Y, Jung ES, Kim MS. A lightweight deep learning model for fast electrocardiographic beats classification with a wearable cardiac monitor: Development and validation study. JMIR Med Informatics 2020; [doi: 10.2196/17037]

3. Chen E, Jiang J, Su R, Gao M, Zhu S, Zhou J, Huo Y. A new smart wristband equipped with an artificial intelligence algorithm to detect atrial fibrillation. Hear Rhythm 2020; [doi: 10.1016/j.hrthm.2020.01.034]

4. Xia Y, Xie Y. A novel wearable electrocardiogram classification system using convolutional neural networks and active learning. IEEE Access 2019; [doi: 10.1109/ACCESS.2019.2890865]

5. Patlar Akbulut F, Akan A. A smart wearable system for short-term cardiovascular risk assessment with emotional dynamics. Meas J Int Meas Confed 2018; [doi: 10.1016/j.measurement.2018.06.050]

6. Miao F, Wang X, Yin L, Li Y. A wearable sensor for arterial stiffness monitoring based on machine learning algorithms. IEEE Sens J 2019; [doi: 10.1109/JSEN.2018.2880434]

7. Kim YS, Mahmood M, Lee Y, Kim NK, Kwon S, Herbert R, Kim D, Cho HC, Yeo WH. All-in-One, Wireless, Stretchable Hybrid Electronics for Smart, Connected, and Ambulatory Physiological Monitoring. Adv Sci 2019; [doi: 10.1002/advs.201900939]

8. Xia Y, Zhang H, Xu L, Gao Z, Zhang H, Liu H, Li S. An Automatic Cardiac Arrhythmia Classification System with Wearable Electrocardiogram. IEEE Access 2018; [doi: 10.1109/ACCESS.2018.2807700]

9. Majumder AJA, Elsaadany YA, Young R, Ucci DR. An Energy Efficient Wearable Smart IoT System to Predict Cardiac Arrest. Adv Human-Computer Interact 2019; [doi: 10.1155/2019/1507465]

10. Lee K, Kim S, Choi HO, Lee J, Nam Y. Analyzing electrocardiogram signals obtained from a nymi band to detect atrial fibrillation. Multimed Tools Appl 2020; [doi: 10.1007/s11042-018-7075-1]

11. Sharma A, Garg N, Patidar S, San Tan R, Acharya UR. Automated pre-screening of arrhythmia using hybrid combination of Fourier–Bessel expansion and LSTM. Comput Biol Med 2020; PMID:32421653

12. Altini M, Casale P, Penders J, Amft O. Cardiorespiratory fitness estimation in free-living using wearable sensors. Artif Intell Med 2016; PMID:26948954

13. Yang C, Aranoff ND, Green P, Tavassolian N. Classification of aortic stenosis using time-frequency features from chest cardio-mechanical signals. IEEE Trans Biomed Eng 2020; PMID:31545706

14. Stehlik J, Schmalfuss C, Bozkurt B, Nativi-Nicolau J, Wohlfahrt P, Wegerich S, Rose K, Ray R, Schofield R, Deswal A, Sekaric J, Anand S, Richards D, Hanson H, Pipke M, Pham M. Continuous wearable monitoring analytics predict heart failure hospitalization: The link-hf multicenter study. Circ Hear Fail 2020; PMID:32093506

15. Pereira T, Ding C, Gadhoumi K, Tran N, Colorado RA, Meisel K, Hu X. Deep learning approaches for plethysmography signal quality assessment in the presence of atrial fibrillation. Physiol Meas 2019; PMID:31766037

16. Kwon S, Hong J, Choi EK, Lee E, Hostallero DE, Kang WJ, Lee B, Jeong ER, Koo BK, Oh S, Yi Y. Deep learning approaches to detect atrial fibrillation using photoplethysmographic signals: Algorithms development study. JMIR mHealth uHealth 2019; [doi: 10.2196/12770]

17. Chou W, Wu PJ, Fang CC, Yen YS, Lin BS. Design of smart brain oxygenation monitoring system for estimating cardiovascular disease severity. IEEE Access 2020; [doi: 10.1109/ACCESS.2020.2997865]

18. Shah AJ, Isakadze N, Levantsevych O, Vest A, Clifford G, Nemati S. Detecting heart failure using wearables: A pilot study. Physiol Meas 2020; PMID:32163936

19. Corino VDA, Laureanti R, Ferranti L, Scarpini G, Lombardi F, Mainardi LT. Detection of atrial fibrillation episodes using a wristband device. Physiol Meas 2017; PMID:28151434

20. Kwon S, Hong J, Choi EK, Lee B, Baik C, Lee E, Jeong ER, Koo BK, Oh S, Yi Y. Detection of atrial fibrillation using a ring-type wearable device (CardioTracker) and deep learning analysis of photoplethysmography signals: Prospective observational proof-of-concept study. J Med Internet Res. 2020. PMID:32348254

21. Lown M, Brown M, Brown C, Yue AM, Shah BN, Corbett SJ, Lewith G, Stuart B, Moore M, Little P. Machine learning detection of atrial fibrillation using wearable technology. PLoS One 2020; PMID:31978173

22. Green EM, van Mourik R, Wolfus C, Heitner SB, Dur O, Semigran MJ. Machine learning detection of obstructive hypertrophic cardiomyopathy using a wearable biosensor. npj Digit Med 2019; [doi: 10.1038/s41746-019-0130-0]

23. Mena LJ, Félix VG, Ochoa A, Ostos R, González E, Aspuru J, Velarde P, Maestre GE. Mobile Personal Health Monitoring for Automated Classification of Electrocardiogram Signals in Elderly. Comput Math Methods Med 2018; PMID:30002725

24. Torres-Soto J, Ashley EA. Multi-task deep learning for cardiac rhythm detection in wearable devices. npj Digit Med 2020; [doi: 10.1038/s41746-020-00320-4]

25. Bashar SK, Han D, Zieneddin F, DIng E, Fitzgibbons TP, Walkey AJ, McManus DD, Javidi B, Chon KH. Novel Density Poincaré Plot Based Machine Learning Method to Detect Atrial Fibrillation from Premature Atrial/Ventricular Contractions. IEEE Trans Biomed Eng 2021; PMID:32746035

26. Inan OT, Baran Pouyan M, Javaid AQ, Dowling S, Etemadi M, Dorier A, Heller JA, Bicen AO, Roy S, De Marco T, Klein L. Novel Wearable Seismocardiography and Machine Learning Algorithms Can Assess Clinical Status of Heart Failure Patients. Circ Heart Fail 2018; PMID:29330154

27. Tison GH, Sanchez JM, Ballinger B, Singh A, Olgin JE, Pletcher MJ, Vittinghoff E, Lee ES, Fan SM, Gladstone RA, Mikell C, Sohoni N, Hsieh J, Marcus GM. Passive detection of atrial fibrillation using a commercially available smartwatch. JAMA Cardiol 2018; PMID:29562087

28. Wasserlauf J, You C, Patel R, Valys A, Albert D, Passman R. Smartwatch Performance for the Detection and Quantification of Atrial Fibrillation. Circ Arrhythmia Electrophysiol 2019; [doi: 10.1161/CIRCEP.118.006834]

29. Zhao Y, Shang Z, Lian Y. A 13.34 μw Event-Driven Patient-Specific ANN Cardiac Arrhythmia Classifier for Wearable ECG Sensors. IEEE Trans Biomed Circuits Syst 2020; PMID:31794404

30. Alfaras M, Soriano MC, Ortín S. A fast machine learning model for ECG-based heartbeat classification and arrhythmia detection. Front Phys 2019; [doi: 10.3389/fphy.2019.00109]

31. Wu J, Li F, Chen Z, Pu Y, Zhan M. A Neural Network-Based ECG Classification Processor with Exploitation of Heartbeat Similarity. IEEE Access 2019; [doi: 10.1109/ACCESS.2019.2956179]

32. Tang X, Ma Z, Hu Q, Tang W. A Real-Time Arrhythmia Heartbeats Classification Algorithm Using Parallel Delta Modulations and Rotated Linear-Kernel Support Vector Machines. IEEE Trans Biomed Eng 2020; PMID:31265382

33. Shao M, Zhou Z, Bin G, Bai Y, Wu S. A wearable electrocardiogram telemonitoring system for atrial fibrillation detection. Sensors (Switzerland) 2020; PMID:31979184

34. Oresko JJ, Jin Z, Cheng J, Huang S, Sun Y, Duschl H, Cheng AC. A wearable smartphone-based platform for real-time cardiovascular disease detection via electrocardiogram processing. IEEE Trans Inf Technol Biomed 2010; PMID:20388600

35. Sadrawi M, Lin CH, Lin YT, Hsieh Y, Kuo CC, Chien JC, Haraikawa K, Abbod MF, Shieh JS. Arrhythmia evaluation in wearable ECG devices. Sensors (Switzerland) 2017; PMID:29068369

36. Ma F, Zhang J, Liang W, Xue J. Automated Classification of Atrial Fibrillation Using Artificial Neural Network for Wearable Devices. Math Probl Eng 2020; [doi: 10.1155/2020/9159158]

37. Gilani M, Eklund JM, Makrehchi M. Automated detection of atrial fibrillation episode using novel heart rate variability features. Proc Annu Int Conf IEEE Eng Med Biol Soc EMBS 2016. PMID:28269045

38. Mei Z, Gu X, Chen H, Chen W. Automatic atrial fibrillation detection based on heart rate variability and spectral features. IEEE Access 2018; [doi: 10.1109/ACCESS.2018.2871220]

39. Zhang H, Dong Z, Gao J, Lu P, Wang Z. Automatic screening method for atrial fibrillation based on lossy compression of the electrocardiogram signal. Physiol Meas 2020; PMID:32464608

40. Huang SF, Lu HP. Classification of temporal data using dynamic time warping and compressed learning. Biomed Signal Process Control 2020; [doi: 10.1016/j.bspc.2019.101781]

41. Rubin J, Parvaneh S, Rahman A, Conroy B, Babaeizadeh S. Densely connected convolutional networks for detection of atrial fibrillation from short single-lead ECG recordings. J Electrocardiol 2018; PMID:30122456

42. Amirshahi A, Hashemi M. ECG Classification Algorithm Based on STDP and R-STDP Neural Networks for Real-Time Monitoring on Ultra Low-Power Personal Wearable Devices. IEEE Trans Biomed Circuits Syst 2019; PMID:31647445

43. Xu X, Liu H. ECG heartbeat classification using convolutional neural networks. IEEE Access 2020; [doi: 10.1109/ACCESS.2020.2964749]

44. Yan Z, Zhou J, Wong WF. Energy efficient ECG classification with spiking neural network. Biomed Signal Process Control 2021; [doi: 10.1016/j.bspc.2020.102170]

45. Wang N, Zhou J, Dai G, Huang J, Xie Y. Energy-Efficient Intelligent ECG Monitoring for Wearable Devices. IEEE Trans Biomed Circuits Syst 2019; PMID:31329129

46. Scirè A, Tropeano F, Anagnostopoulos A, Chatzigiannakis I. Fog-computing-based heartbeat detection and arrhythmia classification using machine learning. Algorithms 2019; [doi: 10.3390/a12020032]

47. Saadatnejad S, Oveisi M, Hashemi M. LSTM-Based ECG Classification for Continuous Monitoring on Personal Wearable Devices. IEEE J Biomed Heal Informatics 2020; PMID:30990452

48. Lui HW, Chow KL. Multiclass classification of myocardial infarction with convolutional and recurrent neural networks for portable ECG devices. Informatics Med Unlocked 2018; [doi: 10.1016/j.imu.2018.08.002]

49. Fan X, Yao Q, Cai Y, Miao F, Sun F, Li Y. Multiscaled Fusion of Deep Convolutional Neural Networks for Screening Atrial Fibrillation from Single Lead Short ECG Recordings. IEEE J Biomed Heal Informatics 2018; PMID:30106699

50. Feng K, Pi X, Liu H, Sun K. Myocardial infarction classification based on convolutional neural network and recurrent neural network. Appl Sci 2019; [doi: 10.3390/app9091879]

51. Li Y, Pang Y, Wang J, Li X. Patient-specific ECG classification by deeper CNN from generic to dedicated. Neurocomputing 2018; [doi: 10.1016/j.neucom.2018.06.068]

52. Allami R. Premature ventricular contraction analysis for real-time patient monitoring. Biomed Signal Process Control 2019; [doi: 10.1016/j.bspc.2018.08.040]

53. Sopic D, Aminifar A, Aminifar A, Atienza D. Real-Time Event-Driven Classification Technique for Early Detection and Prevention of Myocardial Infarction on Wearable Systems. IEEE Trans Biomed Circuits Syst 2018; [doi: 10.1109/TBCAS.2018.2848477]

54. Kiranyaz S, Ince T, Gabbouj M. Real-Time Patient-Specific ECG Classification by 1-D Convolutional Neural Networks. IEEE Trans Biomed Eng 2016; PMID:26285054

55. Mastoi Q ul ain, Wah TY, Raj RG. Reservoir computing based echo state networks for ventricular heart beat classification. Appl Sci 2019; [doi: 10.3390/app9040702]
